# Photogating Effect of Atomically Thin Graphene/MoS_2_/MoTe_2_ van der Waals Heterostructures

**DOI:** 10.3390/mi14010140

**Published:** 2023-01-04

**Authors:** Do-Hyun Park, Hyo Chan Lee

**Affiliations:** 1 Division of Quantum Phase and Devices, Konkuk University, Seoul 05029, Republic of Korea; 2 Chemical Engineering, Myongji University, Yongin 17058, Republic of Korea

**Keywords:** 2D materials, photodetectors, van der Waals heterostructure

## Abstract

The development of short-wave infrared photodetectors based on various two-dimensional (2D) materials has recently attracted attention because of the ability of these devices to operate at room temperature. Although van der Waals heterostructures of 2D materials with type-II band alignment have significant potential for use in short-wave infrared photodetectors, there is a need to develop photodetectors with high photoresponsivity. In this study, we investigated the photogating of graphene using a monolayer-MoS_2_/monolayer-MoTe_2_ van der Waals heterostructure. By stacking MoS_2_/MoTe_2_ on graphene, we fabricated a broadband photodetector that exhibited a high photoresponsivity (>100 mA/W) and a low dark current (60 nA) over a wide wavelength range (488–1550 nm).

## 1. Introduction

Since the first report of the exfoliation of monolayer graphene [1], many two-dimensional (2D) materials, such as hexagonal boron nitride (hBN) [2], black phosphorous [3], and transition metal chalcogenides, including molybdenum disulfide (MoS_2_) [4] and molybdenum ditelluride (MoTe_2_), have been discovered [5,6]. Various optoelectronic devices, such as optical modulators, photovoltaics, waveguides, light-emitting diodes, and photodetectors, have been fabricated using these 2D materials [7,8,9,10,11,12,13,14]. In particular, significant efforts have been made to fabricate short-wave infrared (SWIR) photodetectors using graphene and other 2D materials [15,16,17,18,19,20,21,22,23,24,25]. These devices are widely used for biological imaging, remote sensing, night vision, and telecommunications, and the use of 2D materials eliminates the need to cool these devices to cryogenic temperatures. To this end, several van der Waals (vdW) heterostructures of 2D materials have been proposed, wherein the 2D materials are held together by vdW forces. Among the most promising vdW heterostructures of 2D materials are those that exhibit type−II band alignment owing to interlayer optical excitation, which allows for infrared absorption even if the bandgaps of the components are too large to allow them to absorb infrared light [26]. Moreover, type-II band alignment promotes charge separation at the interface, which is essential for photodetection [27]. It has recently been demonstrated that a monolayer-MoS_2_/monolayer-MoTe_2_ vdW heterostructure showed a distinct photovoltaic current in the infrared region at room temperature (1550 nm) [28]. However, this photodiode suffered from low photoresponsivity (<0.02 mA/W).

In this study, we investigated the modulation of the charge-carrier density of graphene in a vertical graphene/MoS_2_/MoTe_2_ vdW heterostructure. We fabricated a broadband graphene/MoS_2_ photodetector with a MoS_2_/MoTe_2_ vdW heterostructure as the gate stack. We found that the graphene was significantly doped by the photoexcited charge generated in the MoS_2_/MoTe_2_ heterostructure and that type−II band alignment between MoS_2_ and MoTe_2_ resulted in a photoresponsivity greater than 100 mA/W with a dark current of 60 nA over a wide wavelength range. Thus, we were able to realize a SWIR graphene photodetector with high photoresponsivity.

## 2. Results and Discussion

**Figure 1:**
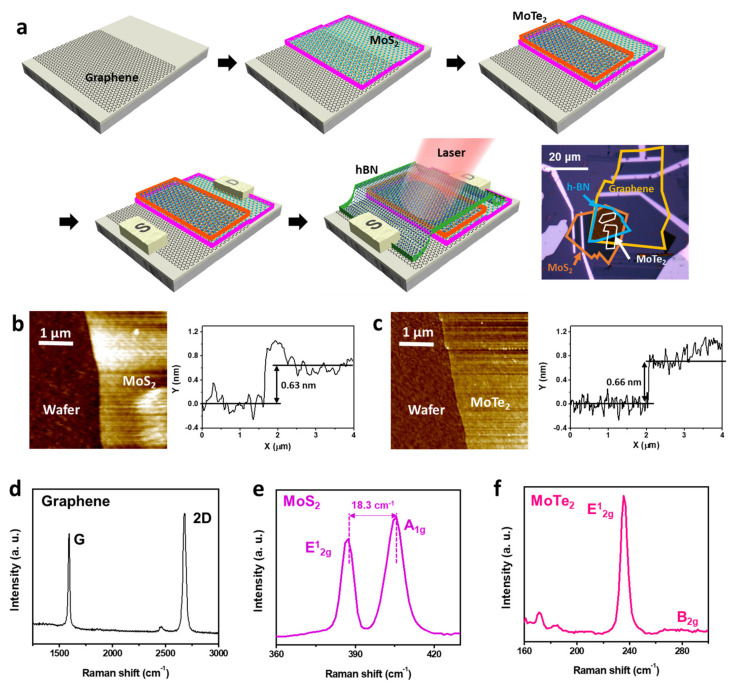
(**a**), Process for fabricating graphene/MoS_2_/MoTe_2_ photodetector. (**b**), AFM images (**left**) and corresponding cross−sectional height profiles (**right**) of standalone MoS_2_ layer and (**c**), MoTe_2_ layer used in photodetector. Raman spectra of (**d**), graphene, (**e**), MoS_2_, and (**f**), MoTe_2_.

Figure 1a shows a schematic of the process for fabricating the photodetector. After monolayer graphene was exfoliated and placed on a wafer substrate, monolayer MoS_2_ was transferred onto the graphene layer using the PMMA transfer method, such that it partially covered the graphene. The overlapping graphene/MoS_2_ region formed the Schottky junction of the device. Next, monolayer MoTe_2_ was transferred onto the graphene/MoS_2_ junction. To passivate the Schottky junction region, we covered it with a thick hBN layer. The vdW heterostructures were then annealed in a vacuum. Finally, we deposited a source electrode on the graphene layer and a drain electrode on the MoS_2_ layer using e−beam lithography. The source and drain electrodes were neither connected to the MoTe_2_ layer nor to the hBN layer. Therefore, the device was a graphene/MoS_2_ Schottky diode in which the MoTe_2_/hBN layer was stacked on the Schottky junction.

Figure 1b,c show atomic force microscopy (AFM) images of the MoS_2_ and MoTe_2_ monolayers on the Si/SiO_2_ wafer used to fabricate the photodetector. The height of the MoS_2_ layer was 0.63 nm and that of the MoTe_2_ layer was 0.66 nm; this confirmed that the MoS_2_ and MoTe_2_ structures were monolayers. To further assess the thicknesses of the graphene, MoS_2_, and MoTe_2_ layers, we determined their Raman spectra before they were transferred (Figure 1d, 532 nm). The ratio of the intensity of the 2D peak (Pos(2D) = 2682 cm^−1^) of graphene to that of its G peak (Pos(G) = 1592 cm^−1^) was slightly larger than 1. The height of the graphene layer was smaller than 1 nm, which indicated that the graphene layer was a monolayer (see Figure S1) [29]. In addition, the D peak of graphene conventionally observed at approximately 1350 cm^−1^, whose intensity is proportional to the defect density of graphene, was not present [30]. The two peaks of MoS_2_ seen at 387.4 and 405.7 cm^−1^ were the $E_{2g}^{1}$ and $A_{1g}^{}$ peaks, respectively. The distance between these two peaks is indicative of the number of MoS_2_ layers, and it increases as the number of MoS_2_ layers increases [31]. In this study, this distance was ~18 cm^−1^, which confirmed that the MoS_2_ layer was also a monolayer (Figure 1e). Figure 1f shows the Raman spectrum of the MoTe_2_ layer. Only one distinctive peak was present at approximately 240 cm^−1^. This was the $E_{2g}^{1}$ peak of the MoTe_2_. The absence of a peak at approximately 280 cm^−1^ ($B_{2g}^{}$) indicated that this layer was also a monolayer [32].

**Figure 2:**
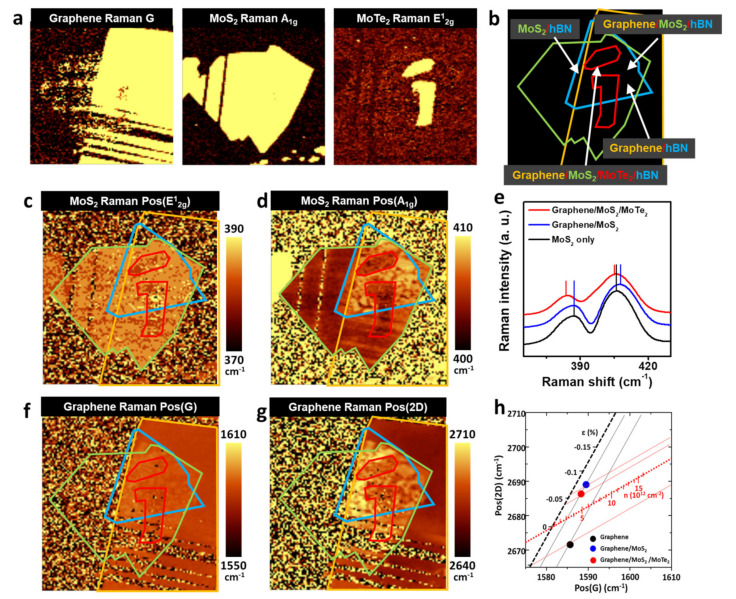
(**a**), Raman intensity maps of G peak of graphene (**left**), A_1g_ peak of MoS_2_ (**middle**), and E^1^_2g_ peak of MoTe_2_ (**right**). (**b**), Locations of graphene (orange), MoS_2_ (green), MoTe_2_ (red), and hBN (blue) as determined using Raman spectroscopy and optical microscopy. (**c**), Pos(E^1^_2g_) and (**d**), Pos(A_1g_) maps of MoS_2_ layer. (**e**), Raman spectra of MoS_2_ in MoS2-only region (black), graphene/MoS_2_ region (blue), and graphene/MoS_2_/MoTe_2_ region (red). (**f**), Pos(G) and (**g**), Pos(2D) maps of graphene. (**h**), Average Pos(G) and Pos(2D) values in Raman spectra of graphene in graphene-only region (black), graphene/MoS_2_ region (blue), and graphene/MoS_2_/MoTe_2_ region (red).

Raman spectroscopy is a powerful tool, not only for the characterization of isolated 2D materials, but also for the vdW stacking of 2D materials, because the Raman active phonon modes of 2D materials are sensitive to changes in the degree of doping and strain of the materials, as well as their vdW interactions with other layers. To evaluate the quality of the vdW stacking at the Schottky junction, we performed a Raman spectroscopy analysis after fabricating the device, as is shown in Figure 1. First, we obtained the Raman intensity maps of the graphene G peak, MoS_2_ A_1g_ peak, and MoTe_2_ E^1^_2g_ peak, which allowed for the delineation of the edges of the graphene, MoS_2_, and MoTe_2_ layers with precision (Figure 2a). Because monolayered MoS_2_ and MoTe_2_ can be degraded by exposure to air, we focused on the MoS_2_, graphene, graphene/MoS_2_, and graphene/MoS_2_/MoTe_2_ regions that were passivated by hBN (Figure 2b). The integration time for one spot in the Raman image was 130 ms, which was sufficiently long for the photoexcited charge carriers in one material to transfer to the other materials. Figure 2c,d show the positions of the MoS_2_ E^1^_2g_ (Pos(E^1^_2g_)) and A_1g_ (Pos(A_1g_)) peaks, respectively. The Raman maps clearly show that the differences in Pos(E^1^_2g_) and Pos(A_1g_) depended on the stacking. Pos(E^1^_2g_) and Pos(A_1g_) were 387.2 and 405.5 cm^−1^, respectively, in the case of the MoS_2_ region; these values were similar to those for MoS_2_ before the transfer process. On the other hand, in the region where MoS_2_ was stacked on graphene, Pos(A_1g_) was blue−shifted by 2 cm^−1^, while Pos(E^1^_2g_) remained the same (Figure 2e). Thus, Pos(E^1^_2g_) and Pos(A_1g_) could be changed by changing the degree of doping [33], strain [34], and vdW interactions with the neighboring materials [35]. However, a shift in the A_1g_ peak of the MoS_2_ by 1 cm^−1^ would require the removal of electrons in a density of 1 × 10^13^. In addition, the biaxial strain in MoS_2_ changes both Pos(E^1^_2g_) and Pos(A_1g_), which was not the case in this study. Therefore, we attributed the blue−shift of the Pos(A_1g_) to the stiffening of the A_1g_ phonon by the graphene−MoS_2_ vdW interaction [35]. This was indicative of interlayer coupling between graphene and MoS_2_. For graphene/MoS_2_/MoTe_2_, the E^1^_2g_ and A_1g_ peaks were red-shifted by ~3 and ~2 cm^−1^, respectively, from those of graphene/MoS_2_, which was consistent with previous reports [36]. We believe that the red-shifting of the peaks was attributable to the tensile strain or the relaxation of the compressive strain owing to the mismatch in the lattice constants of the MoS_2_ (0.316 nm) and MoTe_2_ (0.352 nm).

We also monitored Pos(G) (Figure 2f) and Pos(2D) (Figure 2g) for graphene. Because Pos(G) and Pos(2D) are highly sensitive to the strain and doping level of graphene, these factors can be estimated from the peak positions (Figure 2h) [37]. Pos(G) and Pos(2D) for the graphene−only region were 1585.7 and 2671.5 cm^−1^, respectively; these values correspond to a tensile strain of ~0.05% and hole doping level of ~6 × 10^12^ cm^−2^. However, in the graphene/MoS_2_ region, the G and 2D peaks were both blue-shifted compared with those in the graphene-only region, which indicated that interlayer coupling with MoS_2_ induced a compressive strain of 0.1% and the electron doping of graphene at the ~3 × 10^12^ cm^−2^ level. The compressive strain induced in graphene by MoS_2_ also suggests that the graphene/MoS_2_ heterostructure was well formed in the analyzed area [38]. The electron doping of graphene implies that electrons were transferred from MoS_2_ to graphene. After MoTe_2_ was stacked on graphene, the compressive strain in graphene was relaxed slightly, probably because of the tensile strain generated or the relaxation of the MoS_2_−induced compressive strain by MoTe_2_. In addition, the graphene became less hole-doped compared with the graphene/MoS_2_ region, which meant that the stacking of MoTe_2_ enhanced the transfer of electrons to graphene. The underlying mechanism of graphene doping under the different heterostructures is discussed in more detail later in this paper.

**Figure 3:**
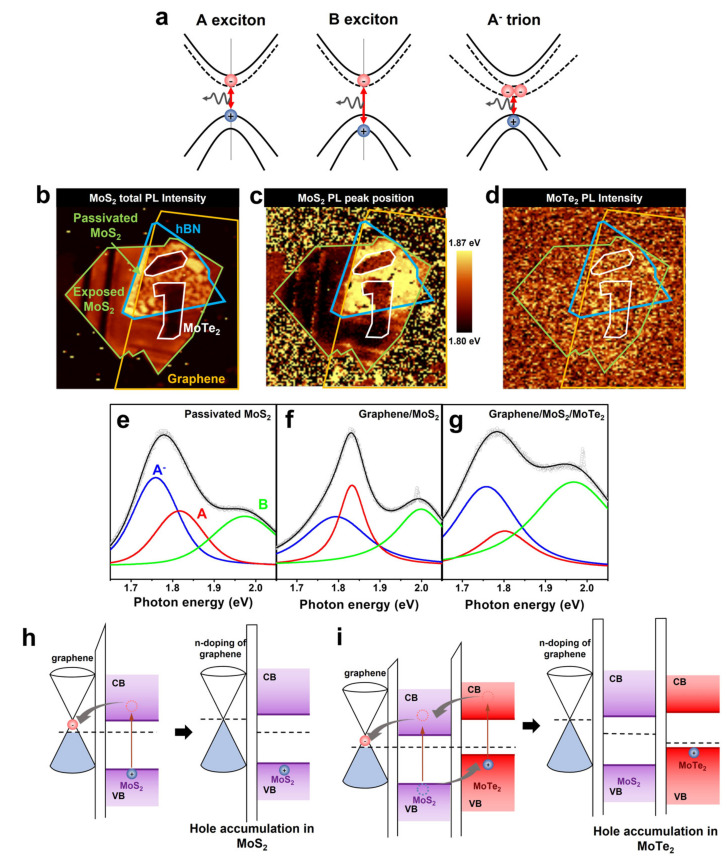
(**a**), Schematics showing A exciton (**left**), B exciton (**middle**), and A− trion (**right**) in MoS_2_. (**b**), MoS_2_ PL intensity map and (**c**), PL peak position map. (**d**), MoTe_2_ PL intensity map. (**e**), PL spectra of MoS_2_ in MoS2-only region (passivated by hBN), (**f**), graphene/MoS_2_ region, and (**g**), graphene/MoS_2_/MoTe_2_ region. (**h**), Energy band diagrams of graphene/MoS_2_, and (**i**), graphene/MoS_2_/MoTe_2_ before (**left**) and under illumination (**right**).

To investigate the charge transfer at the graphene/MoS_2_ and MoS_2_/MoTe_2_ interfaces, we performed photoluminescence (PL) spectroscopy (Figure 3). The PL spectrum of monolayered MoS_2_ originates from the radiative recombination of three types of quasiparticles: A excitons (~1.85 eV), B excitons (~2.03 eV), and A− trions (~1.80 eV) [39]. Figure 3a shows the band diagrams of the three quasiparticles. The relative contributions of the A excitons and A− trions to the PL spectrum of MoS_2_ depend on the Fermi level of MoS_2_ [40]. When MoS_2_ is hole-doped or less electron-doped, the PL peak from the A excitons is very strong compared with that of the A− trions. On the other hand, the A exciton peak decreases as MoS_2_ becomes n-doped, because an excessive number of electrons in MoS_2_ bind to the photoexcited electron-hole pairs to form trions.

Figure 3b,c show the total PL intensity and position of the A peak (A exciton + A− trion, respectively). The efficient PL quenching of graphene differentiates the graphene/MoS_2_/MoTe_2_ region from the MoS_2_/MoTe_2_ region. The MoTe_2_ PL peak at approximately 1 eV was not observed, thus confirming that efficient charge separation had occurred between MoS_2_ and MoTe_2_ (Figure 3d).

To further investigate charge transfer at the interfaces, we analyzed the PL spectra of the MoS2-only (Figure 3e), graphene/MoS_2_ (Figure 3f), and graphene/MoS_2_/MoTe_2_ (Figure 3g) regions in the 1.70–2.05 eV range. The PL spectrum of the MoS2-only region exhibited A exciton, B exciton, and A− trion PL peaks (Figure 3e). Specifically, the A− trion peak was stronger than the A exciton peak, which was in keeping with the fact that MoS_2_ is an n-type semiconductor. The PL spectrum of MoS_2_ changed when it was placed over graphene. In this case, the exciton PL peak was stronger than the A− trion peak. This means that the photoexcited electrons in MoS_2_ were transferred to graphene, while the photoexcited holes were accumulated in MoS_2_. This charge transfer can inhibit the radiative recombination of A− trions in MoS_2_ by spatially separating the photogenerated electrons and holes. In addition, it simultaneously induced the electron doping of graphene during the optical measurements. This was consistent with the Raman spectroscopy analysis, which showed that the formation of the graphene/MoS_2_ structure resulted in the n-type doping of graphene (Figure 2h). The work function of the MoS_2_ was larger than that of graphene [41]. Thus, the electrons in the graphene were transferred to the MoS_2_ layer after contact, resulting in an electric field whose direction was toward MoS_2_ at the graphene/MoS_2_ interface, as is shown in Figure 3h. Therefore, the photogenerated electrons in MoS_2_ could be easily transferred to graphene.

In the region where MoTe_2_ was stacked on the graphene/MoS_2_ structure, resulting in graphene/MoS_2_/MoTe_2_, the A exciton peak was not observed. This implies that holes did not accumulate in the MoS_2_ layer in the resulting structure. This can be explained by the fact that MoS_2_ and MoTe_2_ exhibited type-II band alignment (Figure 3i) and that the photogenerated holes (electrons) in MoS_2_ (MoTe_2_) were transferred to MoTe_2_ (MoS_2_). The transfer of holes from MoS_2_ to MoTe_2_ aided the separation of the photoexcited electrons and holes in graphene, resulting in efficient PL quenching (Figure 3b). The type−II band alignment between MoS_2_ and MoTe_2_ also explains the increased electron doping of graphene in graphene/MoS_2_/MoTe_2_ after the stacking of MoTe_2_ on graphene/MoS_2_, as per the Raman spectroscopy analysis (Figure 2h). The photoexcited holes trapped in MoTe_2_ could enter graphene through MoS_2_, resulting in the additional electron doping of graphene. This is because the MoS_2_ layer was too thin to screen for holes in MoTe_2_.

**Figure 4:**
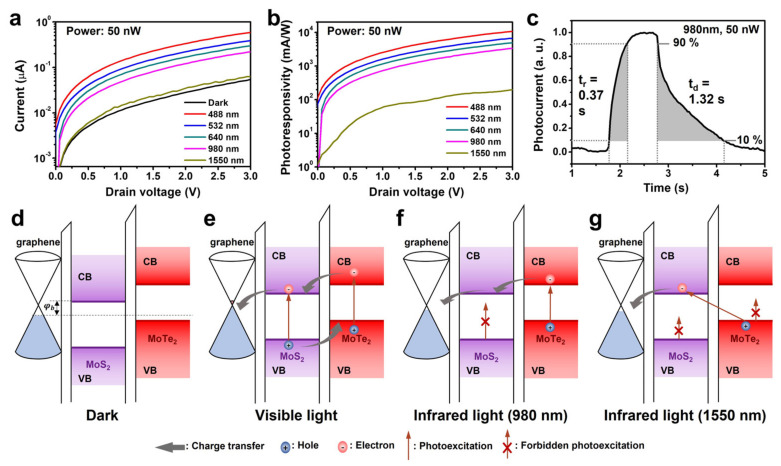
(**a**), Current-voltage curves of photodetector under illumination with 50 nW laser and (**b**), corresponding photoresponsivity-voltage curves. (**c**), Time-resolved photocurrent of device under illumination with 50 nW laser at 980 nm. (**d**), Energy band diagram and charge-transfer processes in dark and under illumination with (**e**), visible laser, (**f**), infrared laser at 980 nm, and (**g**), infrared laser at 1550 nm.

Figure 4a shows the current-voltage (*I*–*V*) characteristics of the photodetector under continuous illumination with a 50 nW light over the graphene/MoS_2_/MoTe_2_ region. The laser spot was placed away from the metal electrodes to exclude the photocurrent from the graphene/metal and MoS_2_/metal junctions. In the dark, the *I*–*V* characteristics were similar to those of a typical Schottky diode with series resistances (Figure S2). The series resistances can be attributed to the resistances of the graphene and MoS_2_ layers and the contact resistances between graphene, MoS_2_ layers, and the metal electrodes. The current increased under illumination, whose wavelength range was 488–1550 nm. A finite photocurrent was observed at zero voltage, showing the photovoltaic effect of the device [42]. However, the photocurrent was almost absent at zero voltage and increased as *V* was increased. It implies that the photocurrent mainly originated from the photogating effect rather than the photovoltaic effect.

For photodetectors based on 2D materials, the photoresponsivity, *R*, and specific detectivity, D*, are generally used as the figures of merit [42]. *R* is the photocurrent per incident unit optical power, and D* is a measure of the smallest detectable signal from the photodetector and is given by $D^{*}=RA^{1/2}/{\bar{I_{n}{}^{2}}}^{1/2}$, where *A* is the illumination area (1200 μm^2^) and ${\bar{I_{n}{}^{2}}}^{1/2}$ is the root-mean-square noise current. The area of the whole photodetector was larger than the laser spot size. Figure 4b shows the estimated *R* value as a function of *V* for various wavelengths. The device exhibited the maximum *R* value at *V* = 3 V; however, *R* increased when we applied a higher *V*. For instance, *R* exceeded 10^4^ mA/W at 488 nm and 10^2^ mA/W at all the other wavelengths. Notably, *R* was 3 × 10^3^ and 1.8 × 10^2^ mA/W at 980 and 1550 nm, respectively; these are the wavelengths at which MoS_2_ is optically inactive. When the noise current is dominated by shot noise, D* can be estimated using the following equation [43]:

(1) $$D^{*}=RA^{1/2}/{\left(2eI_{dark}\right)}^{1/2}$$

D* was estimated to be 3 × 10^11^, 9 × 10^10^, and 5 × 10^9^ Jones at 488, 980, and 1550 nm, respectively. Figure 4c shows the time-resolved photocurrent of the device under illumination with a 980 nm light at *V* = 3 V. The rise and decay times were 0.37 and 1.32 s, respectively (see also Table S1 [15,22,24,44,45,46,47,48,49]).

In the present study, the photogating effect was a photoinduced change in the Fermi level of the material in question, namely, the graphene under MoS_2_. Under the photogating effect, the value of $\varphi_{b}$ at the graphene/MoS_2_ junction was modulated by light, which, in turn, changed the device current. The photogating of the graphene-neighboring MoS_2_ layer was well known (Figure 4d,e) [50]. The MoS_2_ layer absorbed light, resulting in the photoexcitation of electrons and holes. Owing to the electric field at the graphene/MoS_2_ interface and the difference in the energies of the conduction band edge of MoS_2_ and the Fermi level of graphene, the photoexcited electrons were transferred from MoS_2_ to graphene. Meanwhile, the photoexcited holes of MoS_2_ were transferred to MoTe_2_, where they were trapped. This altered the electric field near the MoS_2_ layer, causing the n-doping of graphene. Consequently, $\varphi_{b}$ decreased under illumination, resulting in a reduction in the Schottky barrier height between graphene and MoS_2_. Although this explains the photoresponse of the device under visible light, the photocurrent under infrared light (980 and 1550 nm) requires an additional explanation, because monolayered MoS_2_ cannot absorb light with wavelengths larger than 800 nm. Considering the MoTe_2_ monolayer, we propose the following mechanism to explain the photoresponse of the device under infrared light. When illuminated with 980 nm light (Figure 4f), MoTe_2_ absorbs the light, generating photoexcited electrons and holes. These photoexcited electrons tunnel toward graphene directly or through the MoS_2_ layer, while the photoexcited holes remain in MoTe_2_. This leads to the n-doping of graphene, resulting in a photocurrent in the device. In the case of 1550 nm light (Figure 4g), both MoS_2_ and monolayered MoTe_2_ are optically inactive when they are separated. However, because monolayered MoS_2_ and monolayered MoTe_2_ exhibit type−II band alignment, an interlayer transition occurs between them when they are stacked. The photoexcited electrons in MoS_2_ and holes in MoTe_2_ are separated by the transfer of electrons to graphene or the extraction of electrons owing to the drain bias. The remaining holes in MoTe_2_ cause the n−doping of graphene, thus reducing $\varphi_{b}$ at the graphene/MoS_2_ junction.

To confirm the role of the MoTe_2_ layer, we fabricated a graphene/MoS_2_ Schottky diode without a MoTe_2_ layer on the Schottky junction and measured its photoresponse. As shown in Figure S3a, the current of the device barely changed under infrared light (980 and 1550 nm). Time-resolved current measurements were performed under infrared light illumination (Figure S3b). However, no change in the current was observed during the measurements. The absence of a photoresponse excludes the possibility that the detrapping of charge carriers near the graphene/MoS_2_ interface was responsible for the photoresponse under infrared light. Thus, these results clearly indicate that the absorption of infrared light by graphene did not contribute to the photocurrent of the device and that monolayered MoS_2_ alone did not induce the photogating of graphene under infrared light. It is well known that photogenerated charge carriers recombine within a few picoseconds because of plasmon emission and carrier-phonon scattering [51]. Consequently, the photogenerated charge carriers in graphene were annihilated before the charges at the graphene/MoS_2_ interface were separated. In addition, the bandgap of MoS_2_ inhibits the absorption of infrared light, making the graphene/MoS_2_ junction inactive under infrared light illumination. In summary, the observed photoresponse of the photodetector shown in Figure 4 was attributable to the MoTe_2_ layer deposited on the MoS_2_/graphene Schottky junction.

In conclusion, we realized a graphene/MoS_2_ barristor-based photodetector that exploited the photogating of graphene based on the type−II band alignment in the monolayered MoS_2_/monolayered MoTe_2_ structure. The device showed a photoresponsivity as high as 10^4^ mA/W and a detectivity of 3 × 10^11^ Jones under visible light. More importantly, we were able to simultaneously achieve a photoresponsivity of more than 10^2^ mA/W and detectivity of more than 5 × 10^9^ Jones in the 980–1550 nm range.

## Data Availability

Not applicable.

## Supplementary Materials

The following supporting information can be downloaded at: https://www.mdpi.com/article/10.3390/mi14010140/s1, Figure S1: AFM image (left) and corresponding cross-sectional height profile (right) of graphene used in the photodetector; Figure S2: Dark current-voltage characteristics of the photodetector; Figure S3: a, Current–voltage curves of graphene/MoS2 photodetector without MoTe2 layer under illumination with 1-μW laser and b, time-resolved photocurrent of device under illumination with 1-μW laser at 980 nm; Table S1: Comparison of the performance parameters for photodetectors.
