# Supplementary material for: Photogating Effect of Atomically Thin Graphene/MoS2/MoTe2 van der Waals Heterostructures
Source: Micromachines (Basel). 2023 Jan 4;14(1):140. doi: 10.3390/mi14010140 (PMC9866681; doi:10.3390/mi14010140)
Supplement: Supplementary file 1 [file micromachines-14-00140-s001.zip › micromachines-2033684-supplementary.pdf]

micromachines

Article

Photogating effect of atomically thin graphene/MoS_2/$MoTe_2$ van der Waals heterostructures

Do-Hyun Park [1] and Hyo Chan Lee [2,*]

[1] Division of Quantum phase and devices, Konkuk University, Seoul 05029, South Korea
[2] Chemical Engineering, Myongji University, Yongin 17058, South Korea
* Correspondence: hyochan@mju.ac.kr

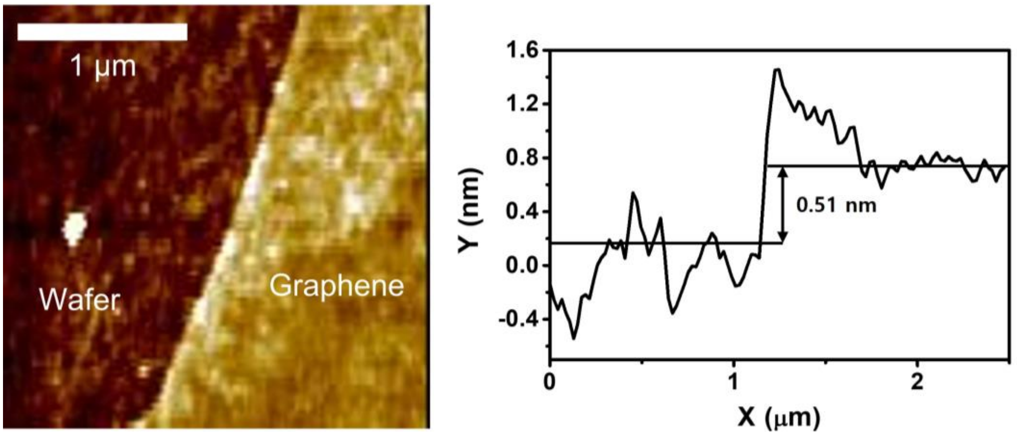

Figure S1. AFM image (left) and corresponding cross-sectional height profile (right) of graphene used in the photodetector.

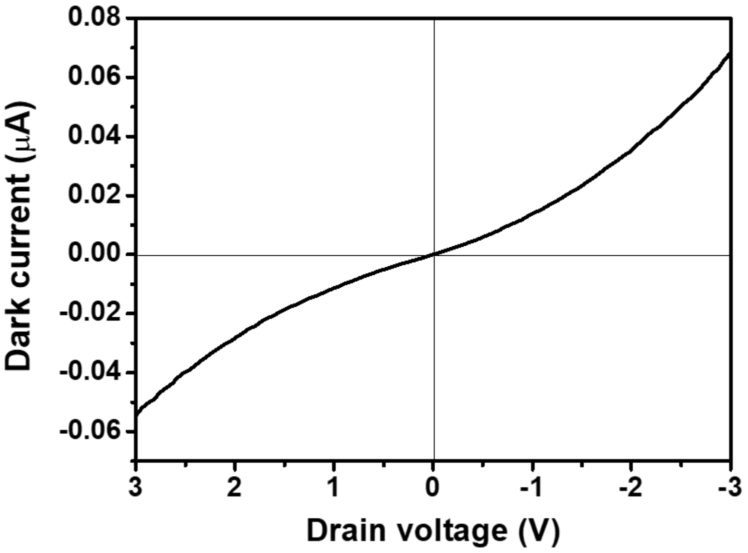

Figure S2. Dark current-voltage characteristics of the photodetector.

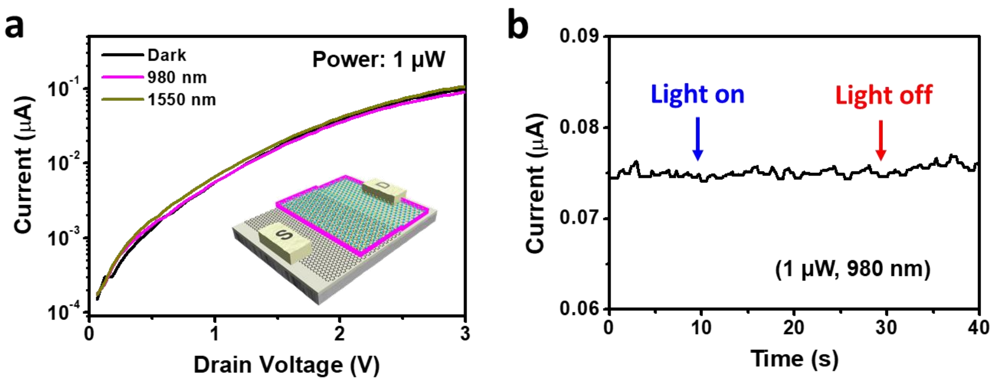

Figure S3. a, Current–voltage curves of graphene/MoS_2 photodetector without $MoTe_2$ layer under illumination with 1-μW laser and **b**, time-resolved photocurrent of device under illumination with 1-μW laser at 980 nm.

Table S1. Comparison of the performance parameters for photodetectors.

Material	Responsivity (R) [A/W]/ Wavelength [nm]	Detectivity(D*) [Jones]	Response time [ms]	Ref.
**$MoTe_2$/MoS_2/ Graphene**	**1.8×10^2 / 1550**	**5×10^9**	**370**	**This work**
Graphene/MoTe/Graphene	0.11 / 1064	-	0.024	[22]
$MoTe_2$/graphene	970.82 / 1064	1.55×10^{11}	78	[15]
Bilayer MoS_2	5.2 / 1070	-	44500	[44]
$MoSe_2$/Si	0.522 / 1100	6.7×10^9	18×10^{-6} (@1550 nm)	[45]
Graphene/Ta_2O_5/Graphene	4 / 1300	-	>500	[46]
Black phosphorus	0.005 / 1550	-	-	[47]
MoS_2/black phosphorus	0.0015 / 1550	2.13×10^9	0.015	[24]
Nitrogen-doped CNT	0.388 / 1550	-	11000	[48]
$PtSe_2$	0.054 / 8350	2.5×10^6	-	[49]
